# Reduced-Gliadin Wheat Bread: An Alternative to the Gluten-Free Diet for Consumers Suffering Gluten-Related Pathologies

**DOI:** 10.1371/journal.pone.0090898

**Published:** 2014-03-12

**Authors:** Javier Gil-Humanes, Fernando Pistón, Rossana Altamirano-Fortoul, Ana Real, Isabel Comino, Carolina Sousa, Cristina M. Rosell, Francisco Barro

**Affiliations:** 1 Instituto de Agricultura Sostenible, CSIC, Córdoba, Spain; 2 Instituto de Agroquímica y Tecnología de Alimentos, CSIC, Valencia, Spain; 3 Departamento de Microbiología y Parasitología, Facultad de Farmacia, Universidad de Sevilla, Sevilla, Spain; Tulane University, United States of America

## Abstract

Wheat flour cannot be tolerated by those who suffer allergies to gluten. Human pathologies associated with grain proteins have increased worldwide in recent years, and the only effective treatment available is a lifelong gluten-free diet, which is complicated to follow and detrimental to gut health. This manuscript describes the development of wheat bread potentially suitable for celiac patients and other gluten-intolerant individuals. We have made bread using wheat flour with very low content of the specific gluten proteins (near gliadin-free) that are the causal agents for pathologies such as celiac disease. Loaves were compared with normal wheat breads and rice bread. Organoleptic, nutritional, and immunotoxic properties were studied. The reduced-gliadin breads showed baking and sensory properties, and overall acceptance, similar to those of normal flour, but with up to 97% lower gliadin content. Moreover, the low-gliadin flour has improved nutritional properties since its lysine content is significantly higher than that of normal flour. Conservative estimates indicate that celiac patients could safely consume 67 grams of bread per day that is made with low-gliadin flour. However, additional studies, such as feeding trials with gluten-intolerant patients, are still needed in order to determine whether or not the product can be consumed by the general celiac population, as well as the actual tolerated amount that can be safely ingested. The results presented here offer a major opportunity to improve the quality of life for millions of sufferers of gluten intolerance throughout the world.

## Introduction

Wheat is a major component of most worldwide diets because of its nutritional quality, and the ability of its flour to produce a variety of tasty and satisfying foods. This is a consequence of the unique viscoelastic properties of wheat dough, which allow the entrapment of CO_2_ during fermentation, enabling the preparation of leavened breads and other baked products. These wheat products make substantial contributions to the dietary intake of energy and protein, and supply dietary fiber, minerals, vitamins, and phytochemicals [1]. A number of countries recommend consuming 250 g–350 g of bread per day (depending on national food habits), and the World Health Organization (WHO) recommends eating bread several times per day [2]. However, wheat products can have negative impacts on human health for those who experience allergies and intolerances. Three pathologies are associated with gluten intake: a) food allergy to wheat, which affects 0.2–0.5% of the population [3], b) celiac disease (CD), a permanent intolerance to gluten not only from wheat, but also related proteins from rye, barley and some oats, that affects both children and adults at various frequencies [4]–[7], and c) gluten sensitivity, a new pathology in which gluten ingestion leads to morphological or symptomatic manifestations despite the absence of CD or wheat allergy [8], with an estimated prevalence of 6% in the USA population. The incidence of these pathologies have increased in recent years both in Europe and the USA, although it is unclear whether this increase can be attributed to the better detection rate, agronomic practices, the use of gluten as a food additive, or breeding for higher protein content [9], [10].

CD is the most studied of the three pathologies and is the result of a complex interaction between genetic and environmental factors. The former is determined by the presence of class II human histocompatibility leukocyte antigen (HLA) molecules DQ2 or DQ8 in genetically predisposed individuals, whereas the latter is determined by the ingestion, digestion, and subsequent deamidation of certain gluten peptides by tissue transglutaminase (tTG) [11]. In the small intestine, the deamidated gluten peptides can bind directly to the HLA-DQ2 or DQ8 receptors on antigen presenting cells (APCs), and are then presented to gluten-sensitive T-cells leading to the release of cytokines, which eventually causes inflammation reactions resulting in damaged intestinal villi [12]. A delay in diagnosis may cause serious health complications, and even certain types of cancer in prolonged exposures to gluten. Celiac adults present an increased relative risk of suffering non-Hodgkins lymphoma [13], [14], and also other types of gastrointestinal cancer. In addition, CD patients show a 31- to 69-fold increased risk of dying from lymphoma [15], [16], and 18% prevalence of lymphoma as cause of death [17]. The only effective treatment available for CD, as well as for other gluten pathologies, is a lifelong strict gluten-free diet (GFD) [18], [19]. However, a GFD is very complex to follow because gluten is a widespread ingredient in the food industry and consequently dietary transgressions are relatively frequent among CD patients (32–55%) [20]. At the same time, a GFD can be detrimental to gut health as it leads to a reduction in beneficial microbiota and in the ability of faecal residues to stimulate the host’s immunity [21]. Thus, potential alternatives to a GFD are being developed to find new therapies to reduce or eliminate the appearance of symptoms after consumption of gluten-containing foods [22], [23], or to develop new cereals with reduced levels of the immunotoxic epitopes by transgenesis approaches [24]–[27]. Some other studies have reported alternatives to reduce the content of these epitopes in wheat cultivars. These strategies are based on the natural genetic variability found in the *Triticum* ssp. [28]–[30], or in the use of deletion or nullisomic wheat lines [31]–. However, given the high number and complexity of the gliadins genes, and that they are inherited in blocks, the possibility of grouping all the low-toxic gliadin genes in a single variety with good commercial aptitude seems to be a difficult task. Previously, we used an RNA interference (RNAi)-mediated gene silencing approach to down-regulate the content of all α-, γ- and ω-gliadins [25], the gluten proteins in which reside the majority of CD epitopes [34], [35]. Flour of these wheat lines is expected to have low toxicity for CD patients as indicated the reduction in the ability of total gluten proteins to stimulate gluten-specific T-cells isolated from CD patients [25]. In addition, some of these lines presented SDS sedimentation volumes comparable to those of the wild types, indicating a potential good bread-making quality [25], [36]. In the present study, we report that it is possible to produce bread of highly acceptable quality made with flour of these lines. The G12 competitive enzyme-linked immunosorbent assay (ELISA) indicated lower toxicity than normal wheat flour. Physical properties, microstructure, and organoleptic quality are also studied in the *reduced-gliadin* bread. In addition, we also show that flour from these lines has improved nutritional properties due to increased lysine content, which is a desirable trait for improving the human diet, especially in developing countries. This *reduced-gliadin* bread could be extremely important not only for all CD, gluten sensitive and allergic patients to improve their diet, but also to reduce the incidence of all gluten related pathologies, which as in the case of CD, its initiation is associated with the level and duration of exposure to gluten [37], [38].

## Materials and Methods

No permits were required for the described study, which complied with all relevant regulations.

### Plant Material

Four transgenic *reduced-gliadin* lines of *Triticum aestivum* cv. Bobwhite 208 (‘BW208’) and three transgenic *reduced-gliadin* lines of *T. aestivum* cv. Bobwhite 2003 (‘BW2003’), and their respective wild-type lines were assayed using a randomized complete block design with two replicates. Both wild types are spring wheat cultivars obtained by the CIMMYT from the cross CM 33203 with the pedigree Aurora//Kalyan/Bluebird/3/Woodpecker. Cultivar BW2003 has been selected for its high transformation efficiency, and carries the T1BL.1RS translocation from rye. On the other hand, BW208 derives from the SH 98 26 ‘Bobwhite’ line described as highly transformable by Pellegrineschi *et al.* [39], and does not contain the rye translocation. In the present study BW208 and BW2003 showed a total protein content of ∼11.6% and ∼10.5% of dry weight, respectively. The total gluten protein content (gliadins plus glutenins) was about 8.7% and 7.9% of dry weight, respectively for BW208 and BW2003.

All the transgenic *reduced-gliadin* lines were reported previously in [25], and contained the inverted repeat (IR) fragment ω/α (vectors pGhp-ω/α and/or pDhp-ω/α) designed to down regulate all the groups of gliadins by RNAi. Transgenic lines were self-pollinated for 4–5 generations, and they showed normal phenotypes in comparison to their corresponding wild types.

### Grain Milling

White flour was obtained from each of the two independent repetitions of the *reduced-gliadin* and wild-type lines. Grains were hydrated to 16.5% humidity by addition of distilled water in two steps (24 h and 20 h before milling) with continuous shaking. Hydrated seeds (1 kg) of each line were milled separately in two steps in a CD1 Chopin (Chopin Technologies, Villeneuve-la-Garenne Cedex, France) standardized test mill. In the first step white flour and wholemeal flour were obtained. The wholemeal flour was reloaded in a second step of milling, and the resultant white flour was blended with that obtained previously, resulting in a total yield of about 60%. Flour was stored at room temperature (RT) for a week. Commercial rice flour supplied by Harinera Derivats del Blat de Moro, S.L. (Parets del Vallés, Spain) was used to make the gluten-free control bread.

### Bread Baking

Dough was prepared on a flour weight basis: for 300 g flour, 180 ml water (225 ml water for the rice flour), 3.6 g baker’s yeast (Saf-Instant, Lesaffre, France) and 4.8 g table salt were added. Ingredients were mixed in a Farinograph (Brabender GmbH & Co. KG, Germany) for 4 min, and rested for 10 min with a plastic film cover to avoid drying. Dough was divided manually (50 g) and dough pieces were rolled mechanically in a ball homogenizer (Brabender GmbH & Co. KG, Germany). Dough pieces were placed on aluminum trays and fermented for 45 min at 30°C. Dough pieces were baked in an electrical convection oven (Eurofours, Gommegnies, France). The baking process was performed at fixed oven temperature of 180°C for 16 min with 2 initial steam injections of 10 sec each. After baking, bread loaves were rested for 30 min at RT to cool down.

### Bread Characterization

Bread weight and volume were determined in three loaves from each sample. Bread volume was determined by the rapeseed displacement method [40]. Moisture content of the loaves was determined following the ICC Method No. 110/1 [41], with a pre-conditioning step of the bread samples. Three loaves from each sample and their medial slices were scanned (HP Scanjet 4400C, Hewlett–Packard, USA), and height and width were determined to subsequently calculate the width/height ratio. Crust and crumb color was determined by a Chroma Meter CR-400 colorimeter (Konica Minolta Sensing Inc., Japan), and expressed in a CIE-L* a* b* color scale (CIE-Lab). The CIE-Lab color space is composed by three perpendicular axis: L*, a* and b*. These three coordinates indicate the lightness of the color (L*; where *L* = 100 indicates white color and *L* = 0 black color), and its position between green and red (a*; where negative values indicate green and positive values indicate red), and between blue and yellow (b*; where negative values indicate blue and positive values indicate yellow). Two independent measurements were made to each of the three loaves to determine crust and crumb color.

### Descriptive Sensory Analysis

A panel of 11 trained assessors was selected to evaluate the bread samples (n = 20; 10 samples with two repetitions) corresponding to the *reduced-gliadin* and wild-type wheat flours, and the rice flour. The range of experience of the test panelists of participating in descriptive analysis and scale rating of a wide range of bread products varied from 3 to 20 years. All the individuals composing the panel gave their informed consent. The panel evaluated appearance, aroma, flavor, and overall acceptance of each sample in a blind tasting. For evaluation, a set of six samples was presented in slices (1 cm thick) on plastic dishes coded and served in a randomized order. In addition, assessors were provided with mineral water in order to cleanse their palate between tastings. Each assessor received a list of sensorial attributes and their definitions to guide them during the sample evaluation.

### Amino Acids Profile Characterization

Wholemeal flour was used for the characterization of amino acids profile of *reduced-gliadin* and wild-type lines. Flour samples were hydrolyzed using 6 N chloric acid and phenol, and then derivatized and analyzed. For the derivatization we used the AccQ Fluor reagent Kit (Waters). First, 20 µl of the hydrolyzed sample were mixed with 60 µl of buffer solution (0.2 M borate buffer), and afterwards 20 µl of derivatization reagent (2 mg/ml 6-aminoquinolyl-N-hydrosysuccinimidyl carbamate, AQC) was added according to the manufacturer’s instructions. After 10 min at 50°C, the solution was directly injected into the high-performance liquid chromatography tandem mass spectrometry (HPLC-MS/MS) system (Varian 320-MS). The amino acid separation was carried out using 2.5 mM ammonium acetate (pH = 5.75) as solvent A, and a solution of 2.5 mM ammonium acetate (pH = 6) and acetonitrile (30∶70, ammonium acetate:acetronitrile) as solvent B. The Pursuit XRs Ultra 2.8 C18 100×2.0 mm column (Agilent) was used as stationary phase, and the flow was 200 µl/min. The detection was performed by mass spectrometry (MS) with the electrospray ionization mode (ESI) (positive and negative). The amount of amino acid is expressed as percentage of the total sample weight.

### Scanning Electron Microscopy (SEM)

Samples (dough and bread) were frozen and dried, and then fractured manually by using the tip of a razor blade and coated with gold. A JEOL JSM6300 scanning electron microscope (JEOL, Tokyo, Japan) was used to observe the samples at 15 kV at RT. SEM pictures at 1000x and 3000x magnifications were taken to the newly exposed surface of each sample. Samples from lines D894 (reduced gliadin content), E82 (reduced gliadin and low molecular weight (LMW) glutenin content), and the wild type BW208 were analyzed.

### G12 Competitive ELISA

Gluten proteins were extracted according to the manufacturer’s instructions using Universal Gluten Extraction Solution UGES (Biomedal SL, Seville, Spain). Maxisorp microtiter plates (Nunc, Roskilde, Denmark) were coated with Prolamin Working Group (PWG) gliadin solution and incubated overnight at 4°C. The plates were washed with PBS-Tween 20 buffer and blocked with blocking solution (phosphate-buffered saline (PBS)-5% non-fat dry milk) for 1 h at RT. Different dilutions of each sample as well as standard solution of PWG gliadin were made in PBS-bovine serum albumin 3%, to each of which was added horseradish peroxidase–conjugated G12 mAb solution. The samples were pre-incubated at RT and added to the wells. After 30 min of incubation at RT, the plates were washed, and 3,3′,5,5′-tetramethylbenzidine (TMB) substrate solution (Sigma, St. Louis, Missouri, USA) was added. After 30 min of incubation at RT in the dark, the reaction was stopped with 1 M sulfuric acid, and the absorbance at 450 nm was measured (microplate reader UVM340, Asys Hitech GmbH, Eugendorf, Austria). Results were expressed in parts per million (ppm) in dry matter for the flour, and in a 35% humidity basis for the bread loaves. In order to estimate the total gluten content the results obtained were multiplied by two. Data were obtained from three independent experiments with samples run in triplicate.

### Statistical Analysis

Data were analyzed with the statistical software R version 2.12.1 [42] using the Graphical User Interface (GUI) R Commander. Major assumptions of analysis of variance (ANOVA) were confirmed by the Shapiro-Wilk’s test for normal distribution (function ‘shapiro.test’, package stats), by the Levene’s test for homocedasticity (function ‘leveneTest’, package car) and the Ramsey’s regression equation specification error test (RESET) for linearity (‘resettest’; package lmtest), and variables were transformed if required. The statistical analysis between the different lines was carried out by using the analysis of variance (ANOVA) model ‘Variable ∼ Line+Block’ (function ‘aov’, package agricolae), followed by Tukey’s Honestly Significant Difference (HSD) post hoc all-pairwise comparison test (function ‘HSD.test’, package agricolae). In all the statistical analyses P values lower than 0.05 were considered significant.

The differences of the amino acids contents between the control and the low-gluten lines were assessed using the ANOVA model ‘Variable ∼ Block+Line’ with the function ‘lm’ (package stats). Mean comparisons were carried out by Dunnett’s post hoc multiple-comparison test (function ‘glht’, package multcomp). The box-and-whisker plot was plotted with the function ‘boxplot’ (package graphics).

## Results and Discussion

### Physical Characterization of *reduced-gliadin* Bread

**Figure 1:**
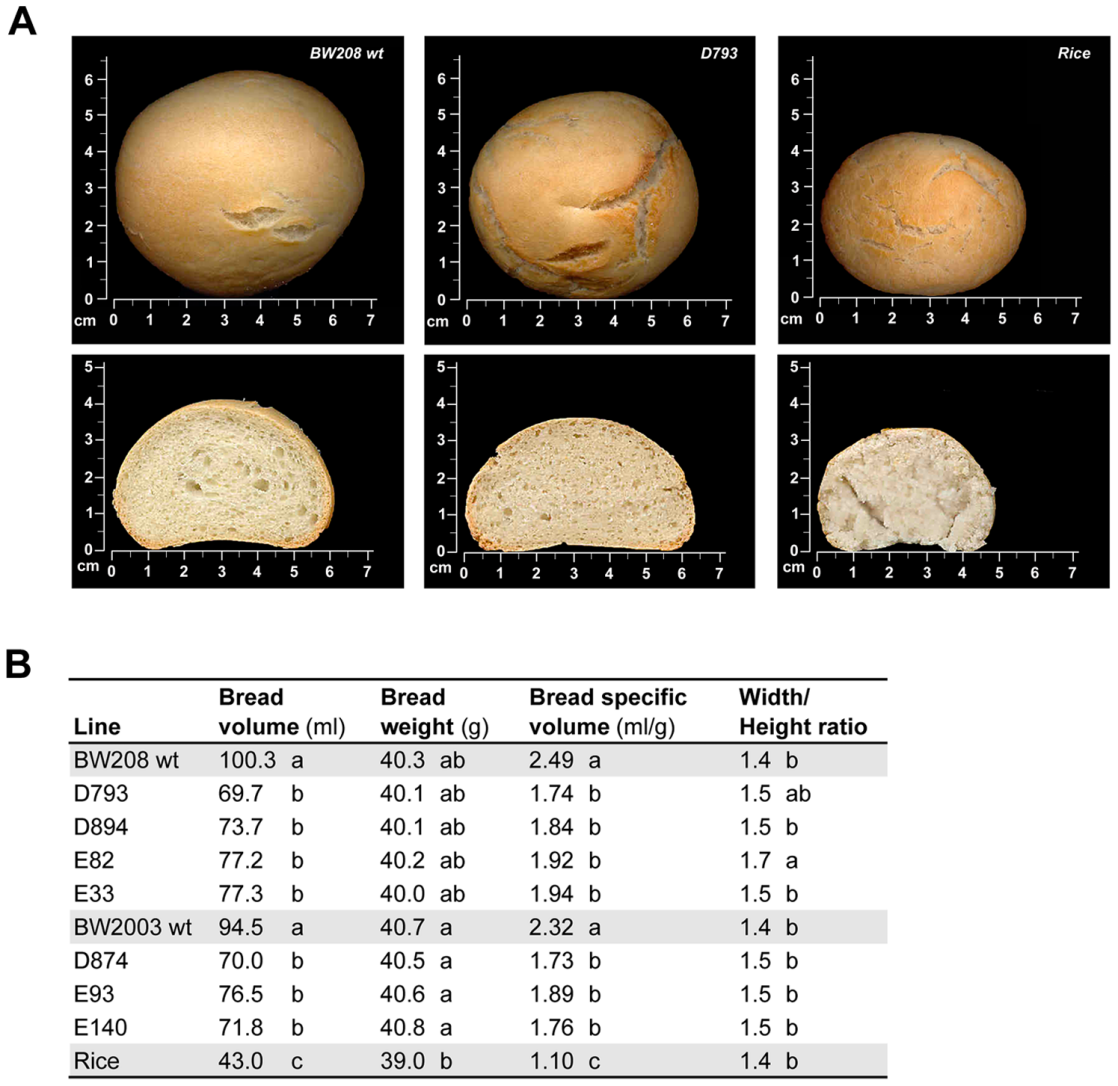
*Reduced-gliadin* bread: physical properties. (A) Loaves and slices of wild-type BW208, *reduced-gliadin* line D793, and rice. (B) Physical properties of bread loaves obtained from wild-type lines, *reduced-gliadin* lines, and rice. Lines with the same letter indicate that no significant differences exist among them as determined by the Tukey HSD post hoc all-pairwise comparison test (P<0.05).

The *reduced-gliadin* breads have been characterized and compared with those from wild-type wheat lines and from rice, as a control of market-typical gluten-free bread. All bread loaves were made by using the same formulation, and following the same mixing and baking methods. The overall visual appearance was very similar in all the wheat-bread loaves of both wild types and *reduced-gliadin* lines (Figure 1A and Figure S1A). The bread slices were cut mechanically, with both the *reduced-gliadin* lines and the wild types presenting similar visual appearance and porous structure, whereas the rice-bread showed a lighter and less porous surface than the wheat counterparts (Figure 1A and Figure S2A). Crust color was very similar in all the samples, whereas crumb showed greater differences between *reduced-gliadin* and wild type lines, and the rice control. The parameter a* (red-green range) was the most variable color parameter (Figures S1B and S2B). Although the shape (ratio width/height) and the weight were comparable in all the wheat loaves independent of the flour source, the volume (ml) and bread specific volume (ml/g) were reduced between 20–30% in the *reduced-gliadin* lines in comparison with the wild types (Figure 1B). This reduction could be explained by a reduced capacity of expansion of the dough during fermentation. Gliadins are known to contribute to the extensibility of the dough [43], and consequently, in the *reduced-gliadin* lines with all the gliadins down-regulated, the three-dimensional network formed by gluten proteins appears to have a reduced extension capacity. Nonetheless, it is sufficient to allow a significant expansion of the dough during fermentation and baking process, producing bread loaves of similar crust and crumb appearance to those made from wild types (Figures S1A and S2A). As reported by Piston *et al.* [36] lines D793, E82, D874, and E93 also presented down-regulation of the LMW fraction; however, none of the physical parameters studied in the present paper were significantly affected by this reduction. Current gluten-free breads available in the market are typically made with rice or maize flour, and although they represent economical ingredients, in general the loaf quality, shelf-life and organoleptic properties are substantially reduced compared to the wheat-flour counterparts [44]. Furthermore, the substitution of food with gluten-free alternatives may result in inadequate intakes of important nutrients since the nutritional properties (protein, fiber, and essential micronutrients) of gluten-free breads are frequently reduced [45], producing nutritional deficiencies in patients suffering gluten-related pathologies [46], [47].

### Organoleptic Properties of the *reduced-gliadin* Wheat Bread are Comparable to that of Normal Bread

**Figure 2:**
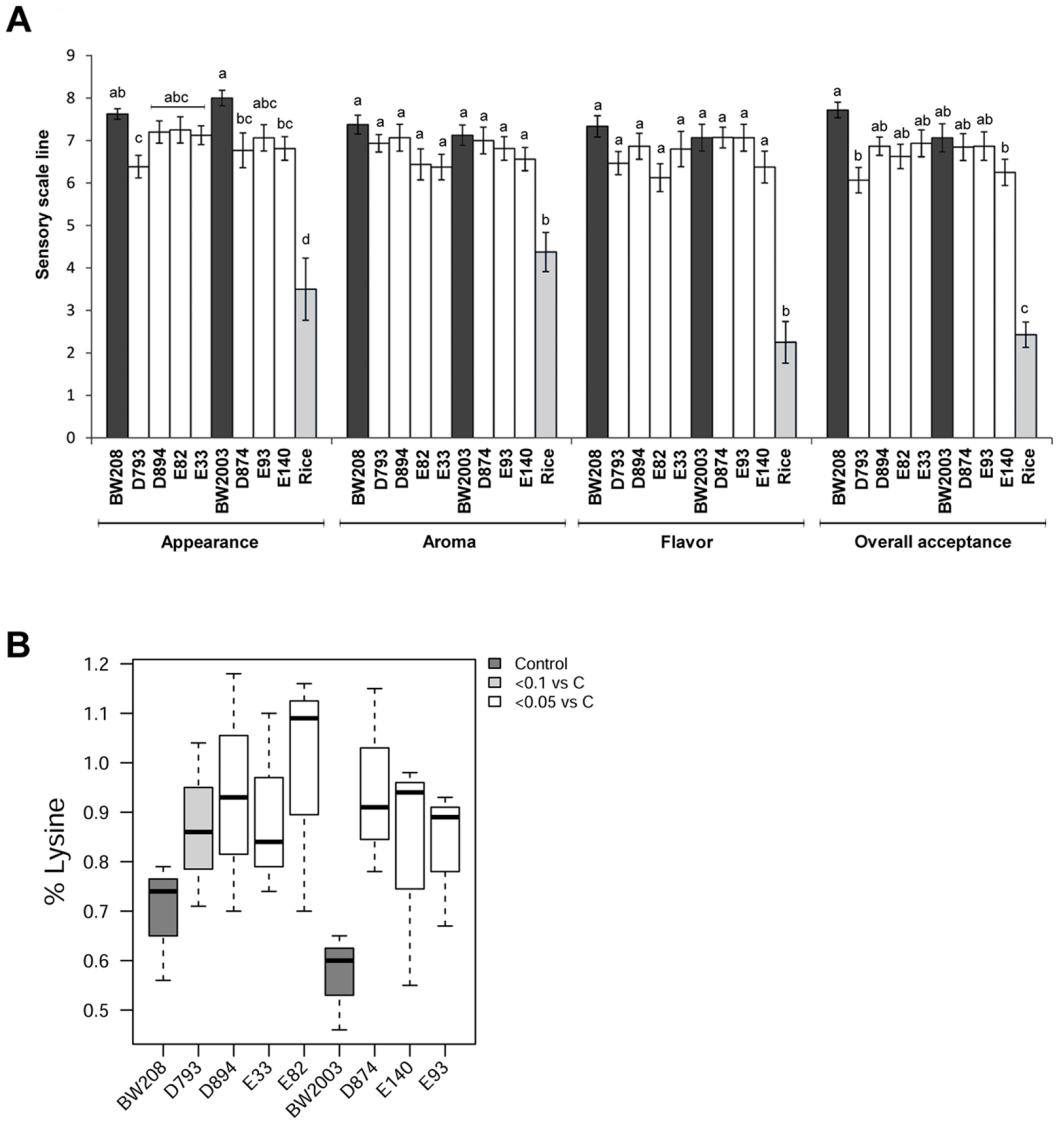
*Reduced-gliadin* bread: organoleptic and nutritional properties. (A) Descriptive sensory analysis. Lines with the same letter indicate that no significant differences exist among them as determined by the Tukey HSD post hoc all-pairwise comparison test (P<0.05). Sensory scale ranges from 1–9. Wheat wild types are represented by black bars, reduced-gliadin lines are represented by white bars, and rice is represented by gray bars. (B) Box-and-whisker plot of lysine content (%) in wild types and reduced-gliadin lines. Dark grey boxes represent the wild-type lines. Significant differences between reduced-gliadin lines and their respective controls are indicated in pale grey (P<0.1), and white (P<0.05).

A descriptive sensory analysis was carried out by a panel of 11 trained assessors, and the scores were expressed in a 1 to 9 hedonic scale (Figure 2A). The rice control presented significantly lower scores than the *reduced-gliadin* and wild-type wheat lines for all the parameters, indicating a higher quality of wheat breads. In addition, most of the *reduced-gliadin* lines showed statistically comparable levels of quality with their wild-type counterparts. Although the overall acceptance was reduced, with an average score of 7.4 for the wild types and 6.6 for the *reduced-gliadin* lines, no significant differences were found between most *reduced-gliadin* lines and wild types. By contrast, the overall acceptance of the rice was significantly lower, presenting a score of 2.4. This indicates, i) there are no differences in terms of quality between these *reduced-gliadin* breads and normal wheat flour breads, and ii) potential consumers would prefer the *reduced-gliadin* bread rather than the rice bread. In addition, a further advantage of the *reduced-gliadin* bread is that it can be made using standard, simple recipes, in contrast to the complex recipes currently used for the manufacture of the rice or maize gluten-free products, which are necessary to give these products an acceptable baking and organoleptic quality [48]–[50].

No differences were found for the organoleptic parameters between the *reduced-gliadin* lines with and without reduction of the LMW fraction. This indicates that, although LMW glutenins play an important role in the bread-making quality, the down-regulation of the LMW glutenins in the lines described here does not affect the overall acceptance of the bread.

### The *reduced-gliadin* Wheat Bread has Increased Lysine Content

Flour samples from *reduced-gliadin* wheat and wild types were compared in terms of amino acid composition (Table S1). Interestingly, the content of lysine was significantly increased in all the *reduced-gliadin* lines (Figure 2B), with increments that ranged between 24–67% respect to the wild types. In wheat, gliadins are known to contain lower amounts of lysine (around 50% less) than glutenins [51]. Consequently, the lysine increase observed in the *reduced-gliadin* lines may be an indirect consequence of the down-regulation of the lysine-poor gliadins and the compensatory increase in other more lysine-rich grain proteins, like the high molecular weight (HMW) glutenins or albumins and globulins, that we have observed in most of these lines [52]. Cereal proteins generally exhibit poor nutritional quality because of a lack of balance in amino acid composition and a low content of lysine. Lysine is considered the most important essential amino acid, and due to it is not synthesized in animals it must be acquired through diet. There is great interest in increasing the content of lysine in cereal crops since it has both an economical and humanitarian importance, especially in developing countries where the diet is mainly composed by a single cereal [53], [54]. Mutant high-Lys lines have been obtained in maize *opaque-2* mutants [55] and *opaque-2*-derived quality protein maize (QPM) lines [56], [57]. Genetic engineering approaches have been also used to increase the lysine content in maize [58], [59] and rice [60]. However, so far there has not been any study describing high-Lys wheat lines. Previous studies with *opaque-2* mutants and QPM maize lines have demonstrated the enhancement of the nutritional properties in animal (rats, pigs, and chickens) [61]–[64] and human nutrition [65], [66], with higher utilizable protein values as a consequence of the lysine increase and a more balanced amino acid composition. Consequently, the increased content of lysine in the wheat lines described in this paper potentially confers an increased nutritive value to the *reduced-gliadin* breads.

### Dough and Bread Crumb Microstructure

**Figure 3:**
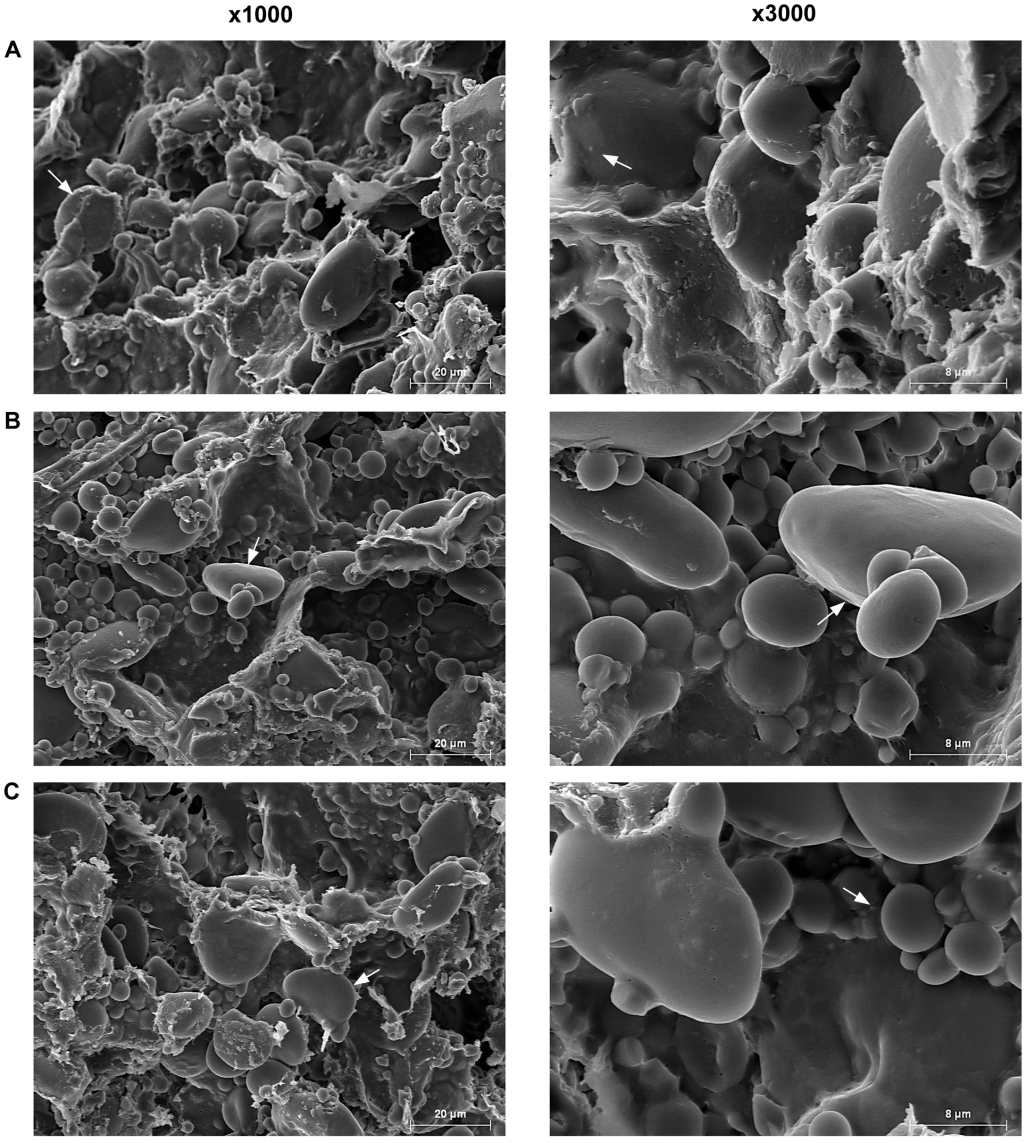
Dough microstructure in *reduced-gliadin* and wild-type wheat lines. SEM was carried out at 1000x and 3000x magnifications in wild type BW208 (A), and *reduced-gliadin* lines E82 (B), and D894 (C). Arrows indicate starch granules covered by gluten in panel (A), and naked starch granules in panels (B) and (C).

Scanning electron microscopy (SEM) was used to study the microstructure of the dough in *reduced-gliadin* and wild-type wheat lines (Figure 3). The micrographs from the wild-type dough (Figure 3A) showed a distinct gluten film surrounding the small and large starch granules. Similar microstructures have been previously observed in wheat doughs [67]. However, in the *reduced-gliadin* lines (Figures 3B and 3C) most of the starch granules appeared naked, especially in the line E82 (Figure 3B). It seems that in the *reduced-gliadin* lines the gluten film is unable to surround all of the starch granules. A possible explanation for this is that the lack of gliadins can be affecting the formation of the gluten structure. It has been previously reported that gliadins may be involved in the development of the structure of the gluten film networks through covalent and non-covalent bonding with other gluten proteins [68]. In addition, some gliadins may be also form inter-molecular disulfide bonds in the gluten [69], stabilizing the gluten film during dough formation. Consequently, the inability of the gluten network to surround the starch granules in the *reduced-gliadin* lines can be explained by either the reduction of gliadins, and/or the increase in the ratio glutenins:gliadins. The microstructure of the bread crumb was also analyzed in the *reduced-gliadin* and the wild-type wheat lines by SEM (Figure S3). In all cases the micrographs showed complex network structures with numerous cavities independently of the line. This network structure corresponds to gluten threads intimately linked to smooth jelly areas resulting from starch gelling, as described previously in [67]. In general the wild types displayed bigger pores than the *reduced-gliadin* lines, as expected given the higher volume of the wild-type breads. In Figure S3 it can be observed that the *reduced-gliadin* line E82 had a similar microstructure to the wild type, with bigger pores than the line D894. In addition, in the line D894 elongated protein strands were more frequently observed.

### Predicted Tolerable Daily Intake of *reduced-gliadin* Bread by Celiac Patients

Finally, the gluten content expressed in parts per million (ppm) of flour and bread samples was determined by a competitive ELISA based on the G12 monoclonal antibody (mAb). The G12 mAb specifically recognizes the hexapeptide QPQLPY present in the 33-mer peptide of α2-gliadin, binding also to related peptide variants found in other immunotoxic gluten proteins [70]. The 33-mer peptide has been identified as one of the digestion-resistant gluten peptides and the main immunodominant toxic peptide in celiac patients [71]. The sensitivity and epitope preferences of G12 antibody was found to be useful for detecting gluten-relevant peptides to infer the potential toxicity of food for patients with CD [72]. The reactivity of G12 mAb with cereal storage proteins of different varieties of cereals was correlated with the immunotoxicity of the dietary grains [73]. We observed that the wild type BW2003 had a higher content of gluten than its counterpart BW208 in both flour and bread samples. In addition, all the *reduced-gliadin* lines showed a large reduction of the immunotoxicity in comparison with their respective wild types, with the BW208 *reduced-gliadin* lines displaying a lower content of gluten than their BW2003 counterparts (Table 1). The amount of gluten was strongly decreased in the bread loaves compared to the flour samples, especially in the *reduced-gliadin* lines. The lines with the strongest depletion of gluten in loaves were line D793 (∼ 97% of reduction) and line E82 (∼ 96% of reduction) (Table 1).

**Table 1:** Gluten content in flour and bread loaves, and estimated maximum tolerable daily intake of bread from wild types and *reduced-gliadin* lines.

	White flour		Bread		
Line	Total gluten ppm (Mean ± SE)	Depletion (%)	Total gluten ppm (Mean ± SE)	Depletion (%)	Tolerable daily intake (g)*
BW208 wt	115042±1000		26616±387		1.9
D793	13073±822	88.6	748±66	97.2	66.9
D894	26860±328	76.7	5349±2061	79.9	9.3
E82	9831±100	91.5	1145.6±299	95.7	43.6
E33	27446±3572	76.1	2933.1±691	89.0	17.0
BW2003 wt	229735±23413		120662±8971		0.4
D874	34095±6674	85.2	11126±1710	90.8	4.5
E93	47495±1412	79.3	12002±1348	90.1	4.2
E140	135786±26500	40.9	62621±3278	48.1	0.8

Gluten content was calculated by G12 competitive ELISA, and is expressed in parts per million (ppm).

*Maximum tolerable daily intake is based in the results reported by Catassi *et al*. [74].

These results were compared with the predicted tolerable daily intake of gluten described by Catassi *et al*. [74]. In that study, a daily intake of 50 mg gluten was established as the maximum dose in order to avoid damage in the small intestine of celiac patients during a prolonged exposure of 90 days. This means that for the wild types the maximum tolerated amount of bread per day would be, in the best of the scenarios, less than 1.9 g. However, for the line E82 a celiac individual could ingest up to 43.6 g per day of bread, and for the line D793 the tolerated amount would be of up to 66.9 g, as determined by the G12 test. In addition, we have previously reported [25] that the immunotoxicity of the remaining gluten in these *reduced-gliadin* wheat lines was up to 100 times lower than the gluten of the wild type wheat, as determined by T-cell assays. Consequently, the above estimations of the tolerated daily intake might be even higher, and still be safe for most of the CD patients. However, in order to determine whether or not the product can be consumed by the general celiac and gluten-intolerant population, as well as the actual tolerated amount that can be safely ingested, additional studies including feeding trials with gluten-sensitive/intolerant patients carrying various combinations of the susceptibility genes are still needed.

## Conclusions

In the present work, the development of wheat bread suitable for celiac patients and other gluten-related pathologies is described. Wheat lines with very low content of the specific gluten proteins (near gliadin-free) that are the causal agents for pathologies such as celiac disease were obtained by RNAi. This *reduced-gliadin* bread has a higher lysine content and similar bread-making quality to normal bread, and therefore could enormously contribute to improve the diet of these patients. Results reported here represent a great advance in the development of food safe for people around the world suffering gluten-related pathologies, with excellent organoleptic properties and greater nutritional quality. However, transgenic wheat is highly regulated and, currently, not commercially grown and this can limit or delay the proposed strategy. The results presented here indicate that flour and bread with reduced levels of these gliadins would be safer for gluten intolerant consumers, although the value of this material still depends on whether or not it can become commercially available, or if these results can be translated into something that is commercially available.

## Supporting Information

Figure S1 **Loaf samples.** (A) Loaves of wild types BW208 and BW2003, *reduced-gliadin* lines, and rice; and (B) color graph indicating the color parameters of wild types, average transgenics (BW208 and BW2003), and rice. The a*, b* and L* values were obtained with a Chroma Meter CR-400 colorimeter, and are represented as fraction of the value respect to the wild-type line BW208.
(TIFF)

Figure S2 **Bread slices.** (A) Slices of wild types BW208 and BW2003, *reduced-gliadin* lines, and rice; and (B) color graph indicating the color parameters of wild types, average transgenics (BW208 and BW2003), and rice. The a*, b* and L* values are represented as described in Figure S1.
(TIF)

Figure S3 **Crumb microstructure of bread samples.** SEM pictures showing the microstructure of the bread crumb in wild-type BW208 (A), and *reduced-gliadin* lines D894 (B) and E82 (C). SEM pictures were obtained at 1000x and 3000x magnifications. Scale bars are shown in each picture.
(TIF)

Table S1 Amino acid content (%) of fresh flour samples of wild types and *low-gliadin* lines.
(DOCX)
