# Supplementary figures and images for: Reduced-Gliadin Wheat Bread: An Alternative to the Gluten-Free Diet for Consumers Suffering Gluten-Related Pathologies
Source: PLoS One. 2014 Mar 12;9(3):e90898. doi: 10.1371/journal.pone.0090898 (PMC3951262; doi:10.1371/journal.pone.0090898)

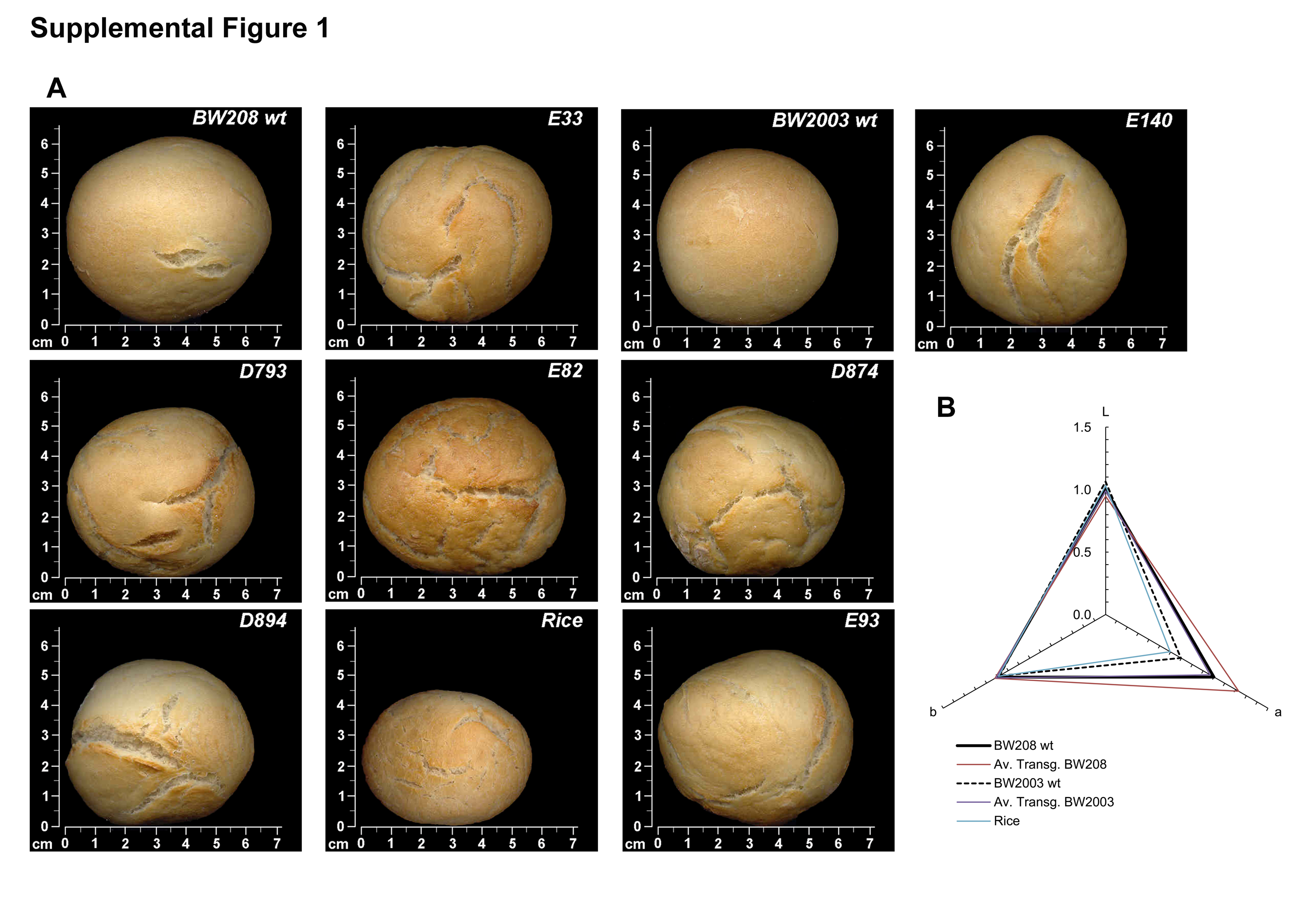

Supplement: Figure S1 — Loaf samples. (A) Loaves of wild types BW208 and BW2003, reduced-gliadin lines, and rice; and (B) color graph indicating the color parameters of wild types, average transgenics (BW208 and BW2003), and rice. The a*, b* and L* values were obtained with a Chroma Meter CR-400 colorimeter, and are represented as fraction of the value respect to the wild-type line BW208. (TIFF) [file pone.0090898.s001.tif]

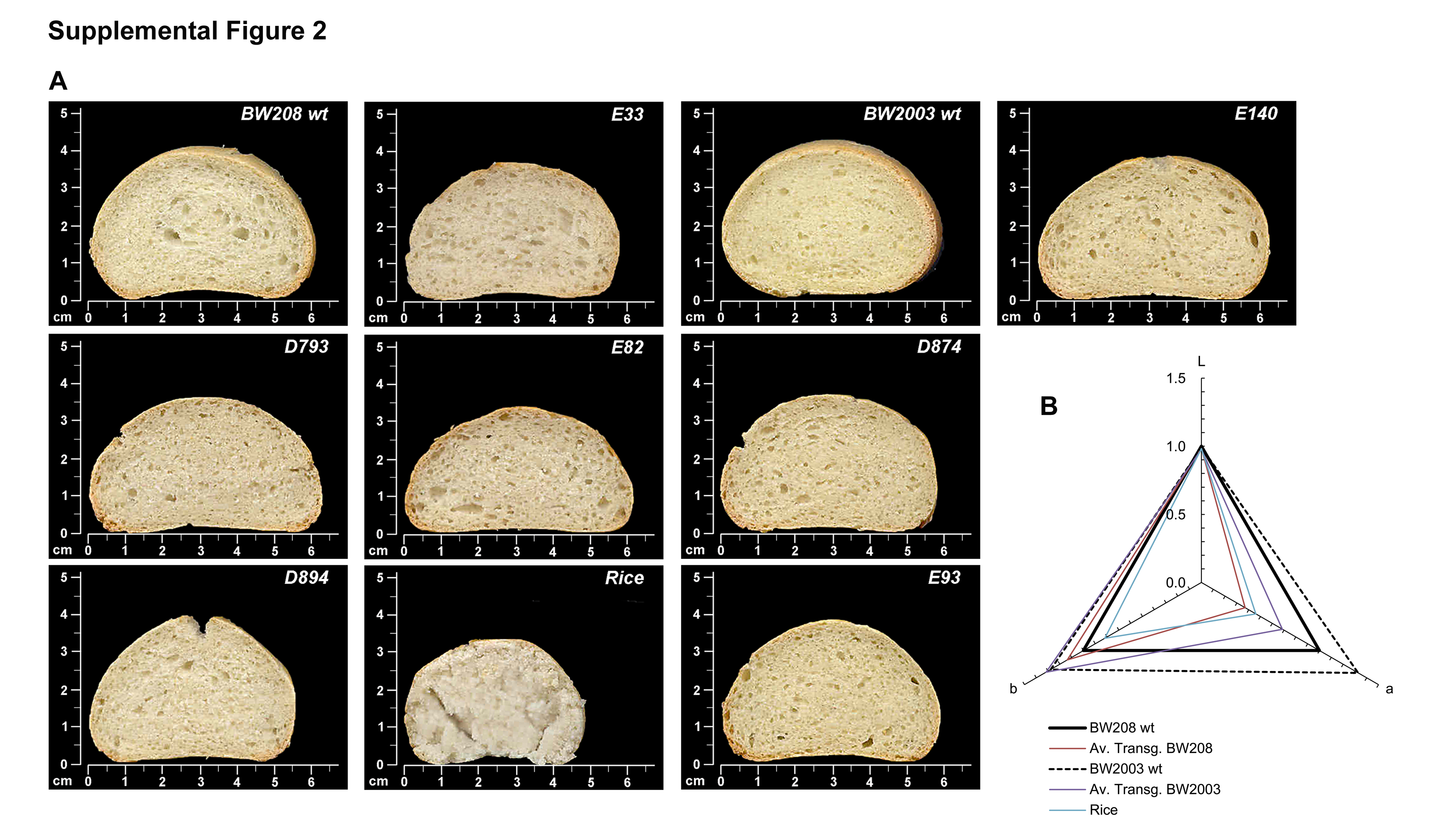

Supplement: Figure S2 — Bread slices. (A) Slices of wild types BW208 and BW2003, reduced-gliadin lines, and rice; and (B) color graph indicating the color parameters of wild types, average transgenics (BW208 and BW2003), and rice. The a*, b* and L* values are represented as described in Figure S1. (TIF) [file pone.0090898.s002.tif]

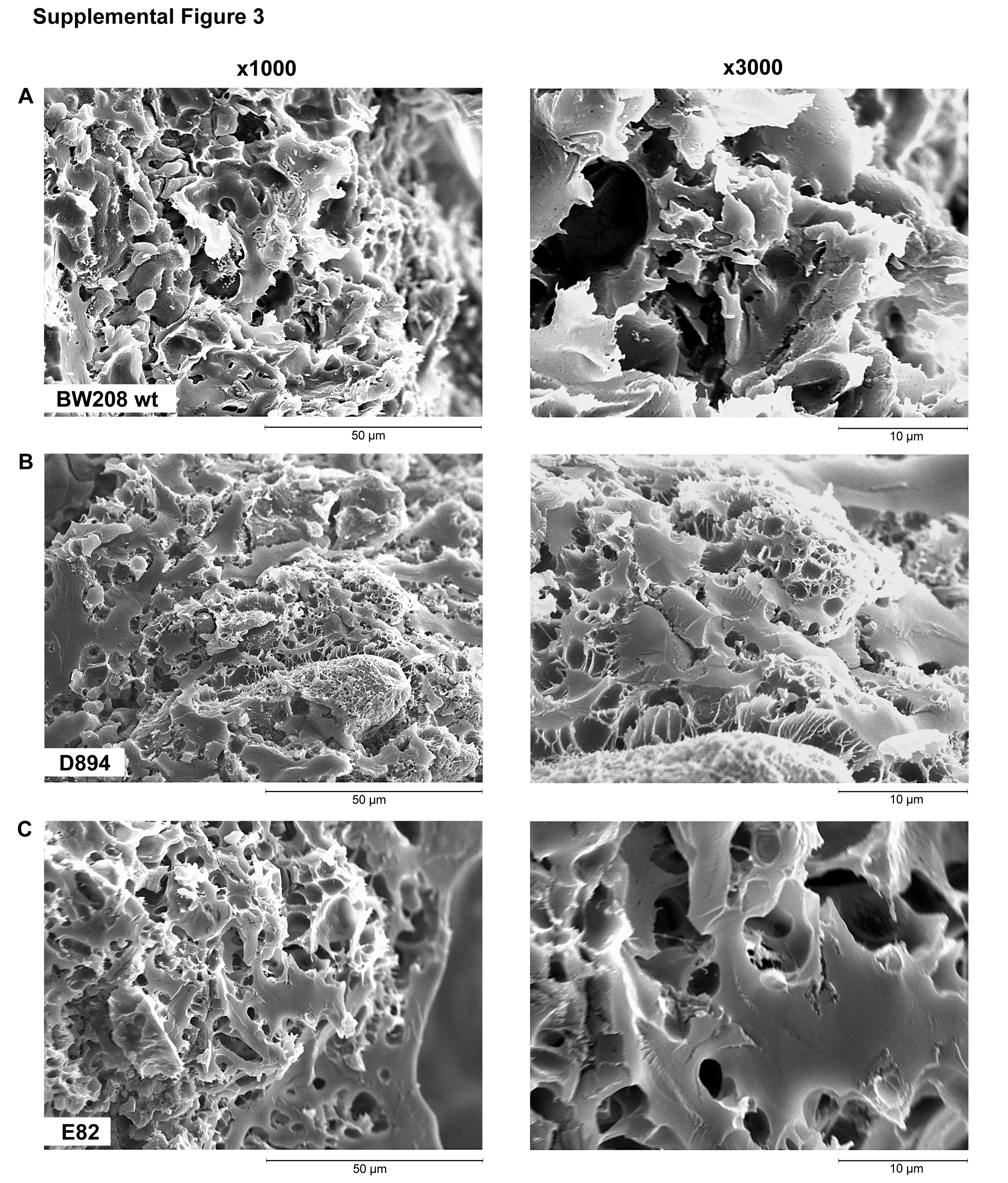

Supplement: Figure S3 — Crumb microstructure of bread samples. SEM pictures showing the microstructure of the bread crumb in wild-type BW208 (A), and reduced-gliadin lines D894 (B) and E82 (C). SEM pictures were obtained at 1000x and 3000x magnifications. Scale bars are shown in each picture. (TIF) [file pone.0090898.s003.tif]
