# Supplementary material for: Reduced-Gliadin Wheat Bread: An Alternative to the Gluten-Free Diet for Consumers Suffering Gluten-Related Pathologies
Source: PLoS One. 2014 Mar 12;9(3):e90898. doi: 10.1371/journal.pone.0090898 (PMC3951262; doi:10.1371/journal.pone.0090898)
Supplement: Table S1 — Amino acid content (%) of fresh flour samples of wild types and low-gliadin lines. (DOCX) [file pone.0090898.s004.docx]

|  |  | **Line** | | | | | | | | |
| --- | --- | --- | --- | --- | --- | --- | --- | --- | --- | --- |
| **Amino Acid** | | **BW208** | **D793** | **D894** | **E82** | **E33** | **BW2003** | **D874** | **E93** | **E140** |
| **Alanine** |  | 0.86 | 0.68 | 0.78 | 0.72 | 0.82 | 0.76 | 0.80 | 0.84 | 0.76 |
| **Arginine** |  | 1.54 | 2.29 | 2.27 | 2.27 | 2.27 | 1.44 | 2.35 | 2.47 | 2.17 |
| **Asparagine** | | *<0.05* | *<0.05* | *<0.05* | *<0.05* | *<0.05* | *<0.05* | *<0.05* | *<0.05* | *<0.05* |
| **Aspartic acid** | | 1.11 | 1.30 | 1.31 | 1.36 | 1.26 | 1.00 | 1.45 | 1.44 | 1.24 |
| **Cysteine** |  | 0.57 | 0.71 | 0.75 | 0.67 | 0.71 | 0.51 | 0.70 | 0.68 | 0.67 |
| **Glutamic acid** | | 4.47 | 2.86 | 3.31 | 2.76 | 3.10 | 4.16 | 3.16 | 3.15 | 3.26 |
| **Glutamine** | | 0.65 | 0.81 | 0.64 | 0.62 | 0.63 | 0.60 | 0.64 | 0.64 | 0.59 |
| **Glycine** |  | 1.16 | 1.22 | 1.39 | 1.07 | 1.33 | 1.18 | 1.31 | 1.54 | 1.28 |
| **Histidine** |  | 0.64 | 0.52 | 0.42 | 0.63 | 0.51 | 0.55 | 0.46 | 0.57 | 0.50 |
| **Isoleucine** | | 0.63 | 0.52 | 0.57 | 0.48 | 0.49 | 0.57 | 0.57 | 0.50 | 0.53 |
| **Leucine** |  | 1.24 | 1.17 | 1.33 | 1.06 | 1.11 | 1.13 | 1.19 | 1.13 | 1.13 |
| **Lysine** |  | 0.70 | 0.87 | 0.94 | 0.98 | 0.89 | 0.57 | 0.95 | 0.83 | 0.82 |
| **Methionine** | | *<0.05* | *<0.05* | *<0.05* | *<0.05* | *<0.05* | *<0.05* | *<0.05* | *<0.05* | *<0.05* |
| **Phenylalanine** | | 0.96 | 0.72 | 0.83 | 0.63 | 0.67 | 0.88 | 0.71 | 0.72 | 0.69 |
| **Proline** |  | 1.80 | 0.82 | 1.16 | 0.87 | 1.09 | 1.66 | 1.04 | 1.18 | 1.22 |
| **Serine** |  | 1.05 | 1.24 | 1.43 | 1.20 | 1.28 | 0.99 | 1.36 | 1.12 | 1.11 |
| **Threonine** | | 0.61 | 0.68 | 0.71 | 0.64 | 0.68 | 0.56 | 0.67 | 0.65 | 0.65 |
| **Tryptophan** | | *<0.05* | *<0.05* | *<0.05* | *<0.05* | *<0.05* | *<0.05* | *<0.05* | *<0.05* | *<0.05* |
| **Tyrosine** |  | 0.82 | 0.99 | 1.00 | 0.78 | 0.85 | 0.82 | 1.03 | 0.98 | 0.87 |
| **Valine** |  | 0.98 | 1.01 | 1.07 | 0.90 | 0.89 | 0.90 | 1.03 | 0.96 | 0.88 |
